# Development of a PrGO-Modified Electrode for Uric Acid Determination in the Presence of Ascorbic Acid by an Electrochemical Technique

**DOI:** 10.3390/s17071539

**Published:** 2017-07-01

**Authors:** Nurulkhalilah Tukimin, Jaafar Abdullah, Yusran Sulaiman

**Affiliations:** 1 Department of Chemistry, Faculty of Science, Universiti Putra Malaysia (UPM), Serdang 43400, Selangor, Malaysia; nurulkhalilah.tukimin@gmail.com (N.T.); jafar@upm.edu.my (J.A.); 2 Institute of Advanced Technology (ITMA), Universiti Putra Malaysia (UPM), Serdang 43400, Selangor, Malaysia

**Keywords:** analytical sensor, uric acid, poly(3,4-ethylenedioxythiophene), reduced graphene oxide, differential pulse voltammetry, electrochemistry

## Abstract

An attractive electrochemical sensor of poly(3,4-ethylenedioxythiophene)/reduced graphene oxide electrode (PrGO) was developed for an electrochemical technique for uric acid (UA) detection in the presence of ascorbic acid (AA). PrGO composite film showed an improved electrocatalytic activity towards UA oxidation in pH 6.0 (0.1 M PBS). The PrGO composite exhibited a high current signal and low charge transfer resistance (*R*_ct_) compared to a reduced graphene oxide (rGO) electrode or a bare glassy carbon electrode (GCE). The limit of detection and sensitivity of PrGO for the detection of UA are 0.19 μM (S/N = 3) and 0.01 μA/μM, respectively, in the range of 1–300 μM of UA.

## 1. Introduction 

Uric acid (UA) is present in human biofluids like urine and blood. It is a protein metabolism byproduct which is also found in large amounts in certain foods that may cause some harm because humans do not have any enzyme to break down uric acid, thus UA will accumulate and contribute to diseases like gout or kidney stones. Gout is a disease where uric acid crystals are formed in the joints which cause a painful inflammatory response [1]. Therefore, the detection of UA is necessary to prevent abnormal levels of UA in the body which can also be related to other kinds of diseases such as hypertension, metabolic syndrome and kidney injury [2].

Poly(3,4-ethylenedioxythiophene) (PEDOT), polyaniline (PANI) and polypyrrole (Ppy) are examples of conducting polymers (CPs) that have been reported as useful materials for making biosensors [3,4] because it shows a high selectivity for analytes in the oxidized state of CP [5]. In sensor technologies, CPs are used as an electrode modification to convey selectivity, to improve the sensitivity, to reduce interferences and as a template for sensor platforms. CP-modified electrodes can detect a lot of different analytes such as ammonia [6], nitrite [7], glucose [4,8] and urea [9]. The properties of CPs such as high conductivity [10], large surface area, low cost and light weight [11] make them appropriate for sensor applications. Yamato et al. [12] and Temmer et al. [13] have reported that poly(3,4-ethylenedioxythiophene) is more suitable for long life biosensor than polypyrrole due to its higher conductivity, high porosity and is less rigid material which allows ions to move rapidly and thus, contribute to a high response rate [13]. In addition, Ppy has a tendency to degrade rapidly at potentials below 400 mV at pH 7 [12].

As a two-dimensional (2D) carbon material, graphene exhibits excellent properties which provide low-cost manufacture, high mechanical strength, good conductivity and high surface area [14,15]. Graphene also has shown excellent electron transfer properties towards lead [16], catechol and hydroquinone [17]. Unfortunately, graphene is hydrophobic because of the π-π interactions that occur between individual layers that cause agglomeration and contribute to its low solubility [18]. However, graphene can be oxidized to form graphene oxide (GO) which can serve as a preferred dopant for the electrochemical and chemical polymerization of conducting polymers due to the existence of an abundance of negatively charged species and it can also increase the solubility of graphene [19]. However, GO has limitations which lead to a low surface area and low conductivity [20]. These problems can be overcome by reducing the GO, forming reduced graphene oxide (rGO) to obtain high electron transfer, high conductivity and good electrochemical activity [21] and when incorporated with PEDOT it produces an excellent biosensor platform for the detection of uric acid. In addition, maintaining the oxygenated groups in rGO at the basal and plane edges allows the formation of bonding with PEDOT [20,22,23].

In this work, PrGO composite was prepared by using a cyclic voltammetry technique. The surface electrochemical properties of PrGO composite were compared with rGO, PEDOT and bare GCE. The electrocatalytic activity of UA oxidation was studied and discussed. The results indicated that PrGO composite was well-suited and well-defined for the fabrication of a UA biosensor in the presence of ascorbic acid (AA) due to its excellent electrochemical performance. PrGO revealed a low detection limit towards oxidation of UA and it also possesses an excellent analytical performance, with good stability, high sensitivity and selectivity for UA. Based on our best knowledge, the use of PrGO composite for the electrocatalytic determination of UA in the presence of AA has not been studied. Thus, this work will focus on the fabrication of a PrGO composite on a GCE substrate for UA detection in the presence of AA by using a differential pulse voltammetry (DPV) technique. The PrGO composites were characterized using Fourier transform infrared (FTIR) spectroscopy, cyclic voltammetry (CV), electrochemical impedance spectroscopy (EIS) and field emission scanning electron microscopy (FESEM).

## 2. Experimental

### 2.1. Reagents and Chemicals

Potassium chloride (KCl) and graphene oxide (GO) were obtained from Graphenea (Gipuzkoa, Spain) and Fisher (Hampton, VA, USA), respectively. Potassium dihydrogen phosphate (KH_2_PO_4_) and dipotassium hydrogen phosphate (K_2_HPO_4_) were supplied by Merck (Kenilworth, NJ, USA). Uric acid (UA), ascorbic acid (AA) and 3,4–ethylene-dioxythiophene (EDOT) were provided by Sigma-Aldrich (St. Louis, MO, USA). Phosphate buffer solution (PBS) was prepared by adjusting the pH value using an appropriate quantity of standard stock solutions of 0.1 M KH_2_PO_4_ and 0.1 M K_2_HPO_4_. Deionized water (18.2 MΩ·cm) from a Milli-Q system (Millipore, Boston, MA, USA) was used to prepare all solutions.

### 2.2. Preparation of PrGO Modified Electrode

Electrodeposition of PrGO was accomplished by using a potentiostat (Autolab M101, Metrohm Autolab, Utrecht, The Netherlands). The GCE was cleaned with alumina slurry (0.5 μm) and sonicated in 1:1 nitric acid (HNO_3_)−distilled water followed by deionized water for 10 minutes. PrGO composite was deposited electrochemically onto the GCE in a solution containing 0.01 M EDOT and 0.01 mg/mL GO solution by using the CV technique between 1.2 V and 1.5 V for three cycles (scan rate: 0.1 V/s).

### 2.3. Instrumentation

The electrochemical measurements were performed using a three-electrode system consisting of a counter electrode (Pt wire), reference electrode (Ag/AgCl) and the working electrode (GCE, Φ = 3 mm). The measurements were performed at room temperature by using a potentiostat (Autolab M101). Cyclic voltammetric scans were applied from −0.2 to 0.6 V (vs. Ag/AgCl). The EIS analyses were carried out at open circuit potentials (OCP) in 5 mM K_3_Fe(CN)_6_/K_4_Fe(CN)_6_ containing 0.1 M KCl with frequency from 10 kHz to 0.1 Hz. The amperometric was performed at 0.5 V. Field emission scanning electron microscopy (FESEM, JSM-7600F, JEOL, Peabody, MA, USA) was applied to examine the morphology of the composites. The presence of functional groups in the PrGO composite was identified by Fourier transform infrared spectroscopy (FTIR, PerkinElmer, Waltham, MA, USA). The pH of the solutions was measured with a pH meter (pH/Ion S220, Merck Millipore, Billerica, MA, USA), which was calibrated with standard buffer solutions.

## 3. Results and Discussion

### 3.1. Electrodeposition

**Figure 1:**
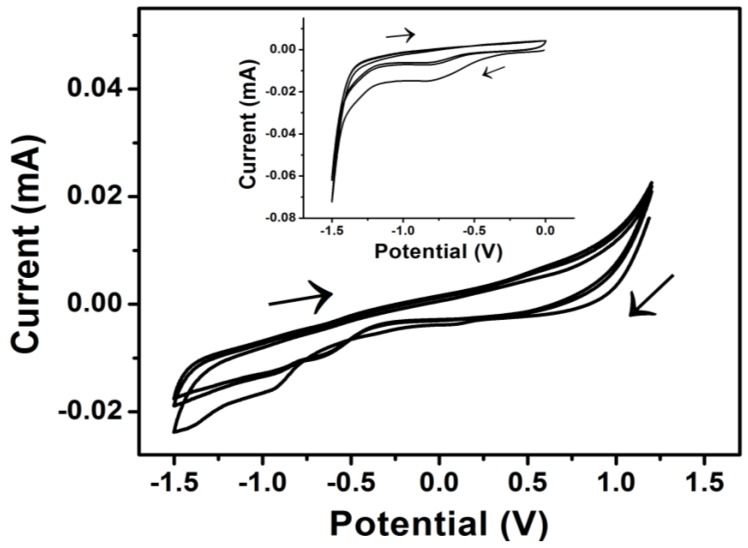
CV of 0.01 mg/mL GO and 0.01 M EDOT on GCE electrode (Scan rate: 0.1 V/s). Inset: CV of GO reduction on GCE at potential 0 to −1.5 V (Scan rate: 0.1 V/s).

The electrodeposition of PrGO was performed by applying 1.2 to −1.5 V in 0.01 mg/mL GO and 0.01 M EDOT for three cycles. The cyclic voltammogram in Figure 1 shows oxidation and reduction peaks at 0.5 V and −0.6 V, respectively, which are due to the polymerization of EDOT [24,25], while the GO reduction peak is observed at around −0.9 V, indicating that the most of the oxygenated groups such as epoxy, carbonyl and hydroxyl groups have been reduced to form rGO [24,26]. The identification of this reduction peak was confirmed by performing CV (inset of Figure 1) in a solution containing GO with the absence of EDOT monomer. However, the reduction potential varies depending on the adsorptivity and reactivity of the different oxygenated functional groups on the GO surface [27,28]. Repetitive cycling of PrGO deposition indicated that as the number of scanning cycles increased, the reductive peak currents decreased which correspond to a reduction of the adsorbed GO on the GCE surface during the second and subsequent cycles [26]. Thus, the CV technique indicates that a significant amount of the oxygenated groups on the GO surface could be reduced by using the electrochemical reduction technique.

### 3.2. Material Characterization

**Figure 2:**
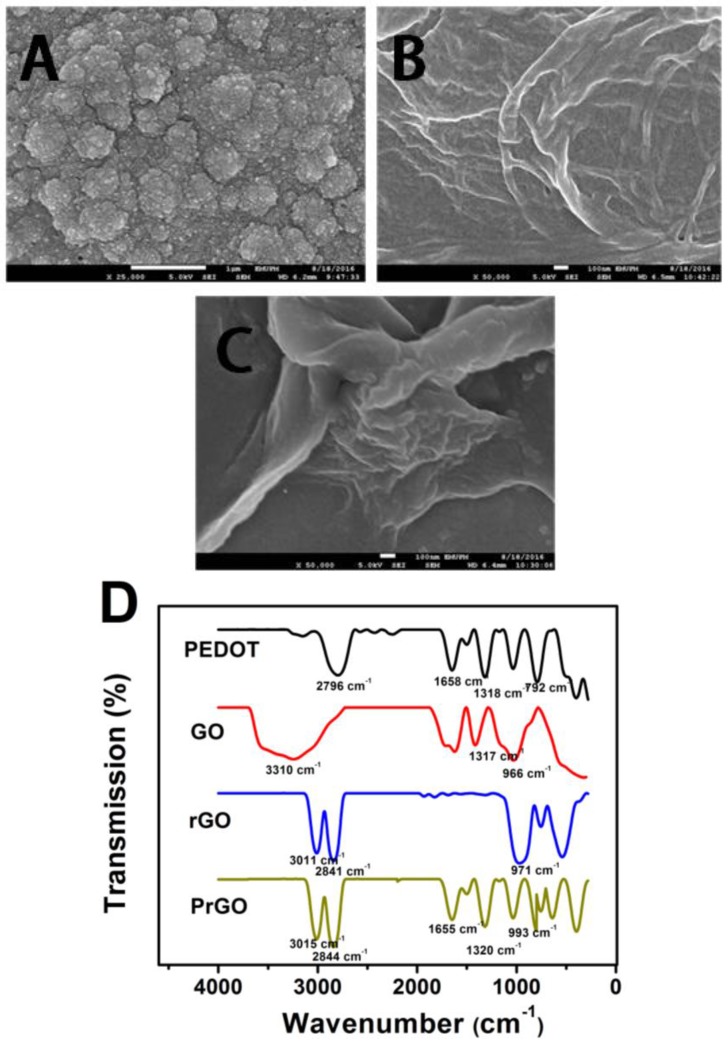
SEM images of (**A**) PEDOT, (**B**) rGO and (**C**) PrGO composite, (**D**) FTIR spectra of PEDOT, GO, rGO and PrGO composite.

SEM images and FTIR spectra of PEDOT, GO, rGO and PrGO composites are shown in Figure 2. PEDOT film (Figure 2A) reveals a granular structure while the rGO film shows wrinkled-like morphology (Figure 2B). The polymerization of PrGO (Figure 2C) results in a very rough surface where PEDOT is coated on the rGO layer surfaces as can be seen in the SEM image. During the electropolymerization of PrGO, the π-π interaction and van der Waals interactions between the aromatic ring of rGO and PEDOT play a significant role to balance the delocalization of ions in both materials [29,30,31] to form PrGO composite. The rGO (dopant) is introduced into PEDOT by electrochemical polymerization which maintaining the charge neutrality and its electrical conductivity is increased [32]. In addition, the synergistic effects of PEDOT and rGO play a significant role in the signal enhancement because of the strength of their interactions which leads to high conductivity. The nature of the rGO as a dopant strongly influences the morphology of the PEDOT [33].

The FTIR spectrum in Figure 2D shows a PEDOT peak at 792 cm^−1^ which is attributed to the C-S stretching mode, whereas, the bands at 1318, 1658 and 2796 cm^−1^ result from the C-O-C, C=O and sp^3^ C-H stretching modes, respectively. The GO spectrum shows peak absorptions assigned to C=O and O-H groups at 1317 cm^−1^ and 3310 cm^−1^, respectively. After the reduction of GO, the intensities of the oxygenated functional group peaks decrease compared to GO. As for PrGO composite, a series of peaks was observed at around 1300, 1600, 2000 and 3000 cm^−1^ which are associated with the functional groups present in PEDOT and rGO film. Hence, it revealed that PEDOT was successfully incorporated with rGO as shown in the SEM image.

**Figure 3:**
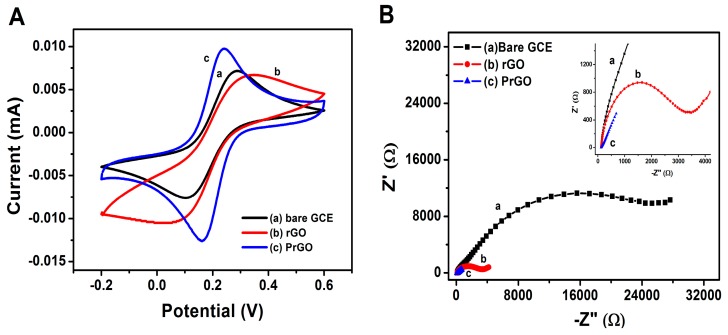
(**A**) CV curves of 1 mM Fe(CN)_6_^3−^ in 0.1 M KCl at (a) bare GCE, (b) rGO and (c) PrGO. Scan rate: 50 mV/s. (**B**) Nyquist plots of 5 mM Fe(CN)_6_^3−/4−^ in 0.1 M KCl at (a) bare GCE, (b) rGO and (c) PrGO. The frequency range is from 0.1 Hz to 100 kHz. The ac amplitude of 5 mV was applied.

Figure 3A shows cyclic voltammograms of three various electrodes in 1 mM K_3_[Fe(CN)_6_] as a redox probe in 0.1 M KCl solution. A redox peak was seen with a separation peak (Δ*E_p_*) of 129 mV for bare GCE, while the Δ*E_p_* values for rGO and PrGO are 178 and 68 mV, respectively. The rGO film possesses larger Δ*E*_p_ than bare GCE and the redox peak currents decreased significantly due to the aggregation of rGO [20] that causes the electrochemical properties to diminish and it becomes electroinactive. Interestingly, the Δ*E*_p_ value for PrGO is smaller than those of bare GCE and rGO, indicating the rate of electron transfer and redox peak current for PrGO is significantly increased which is a result of the excellent electrical conductivity of the combination of PEDOT and rGO films [34]. The electrochemical active surface area (EASA) was then estimated from the peak current (*I*_p_) according to the Randles-Sevcik equation [35]:

$$I_{p}=\left(2.69\times 10^{5}\right)n^{3/2}AD^{1/2}Cv^{1/2}$$

where *n* is the number of electrons taking part in the redox reaction (*n* = 1), A is the electroactive surface area (in cm^2^), *D* is the diffusion coefficient (*D* = 6.5 × 10^−6^ cm^2^·s^−1^), *C* is the bulk concentration (in mol·cm^−3^), *v* is the scan rate (in V·s^−1^). The values of the electrochemically active surface area for bare GCE, rGO and PrGO are calculated as 0.065, 0.046 and 0.100 cm^2^, respectively. The results reveal that the PrGO electrode has larger reaction surface area than the rGO and bare GCE.

EIS is a practical analysis to identify the properties of electron transfer rate occurred on the surface of the electrodes. Figure 3B shows the Nyquist plots of PrGO, rGO and bare GCE in 5 mM K_3_Fe(CN)_6_/K_4_Fe(CN)_6_ (1:1) and 0.1 M KCl. At low frequency, an inclined straight line indicates the diffusion limiting step characteristic for the electrochemical processes [36]. While the high-frequency region consists of the semicircle that correlated to the resistance of charge transfer (*R*_ct_) that lead to the limitation of the electron transfer rate between the redox probe and electrode interface. As seen in Figure 3B, the *R*_ct_ value of rGO (648 Ω) is lower compared to the *R*_ct_ of bare GCE (1202 Ω) due to the conductive properties of the rGO that can accelerate the electron transfer rate. After the modification of the GCE with PrGO, the *R*_ct_ is significantly reduced to 8.23 Ω due to the excellent combination of the conductivity of PEDOT and rGO. The changes in *R*_ct_ indicate that the PrGO composite possesses superior conductivity that enhances the electrocatalytic activity of the electron surface of the PrGO electrode.

### 3.3. Effect of pH and Electrode Stability

**Figure 4:**
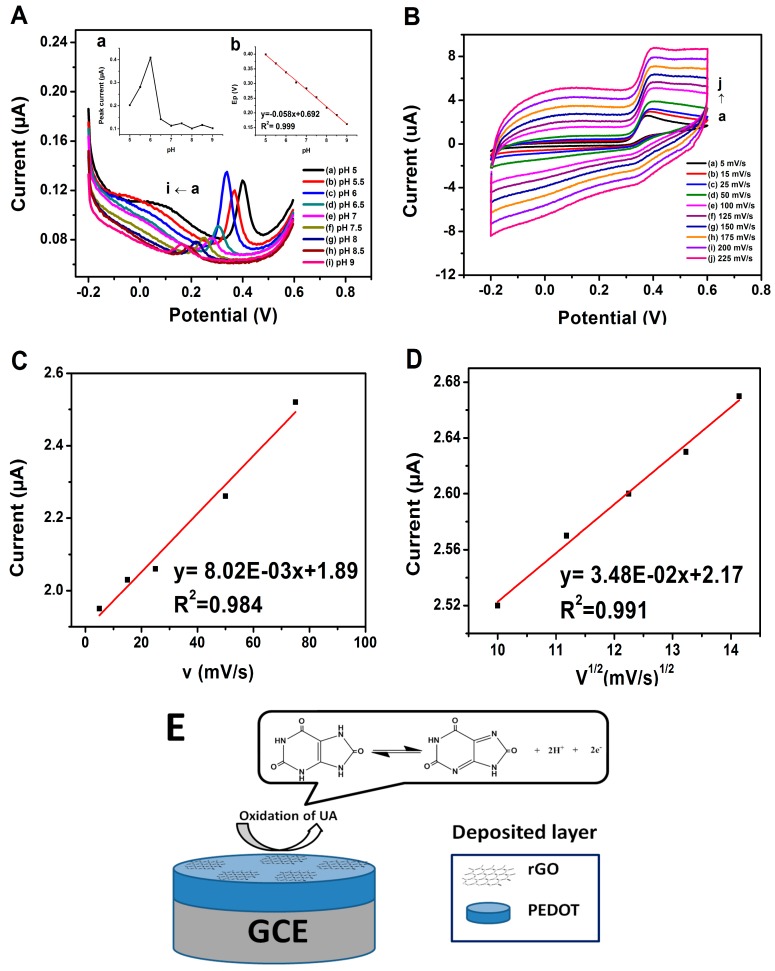
(**A**) DPV of PrGO in 0.1 M PBS of 20 μM of UA at different pH values (pH: 5, 5.5, 6, 6.5, 7, 7.5, 8, 8.5, 9) inset (a) Peak current (*I*_pa_) vs pH (b) Potential (V) vs pH of 0.1 M PBS of 20 μM UA. (**B**) CV of 450 μM UA with various scan rates for PrGO in PBS at pH 6.0. Anodic peak currents (*I*_pa_) as a function of a (**C**) scan rate (**D**) square root of scan rate for the effective working surface area determination. (**E**) Schematic illustration of UA sensing mechanism on PrGO composite.

The important factor that needs to be optimized in the determination of the analyte is the pH of the analyte-containing solution. The pH effect on the determination of 20 μM of UA in 0.1 M PBS at PrGO electrode was observed within the range of 5.0–9.0 by using the DPV method as shown in Figure 4A. 

Hence, as the pH of the PBS solution increased, the peak potential is negatively shifted which demonstrates that protons are involved in the processes of UA oxidation at the PrGO electrode surface [37,38]. The peak potential observed is directly proportional to the pH value according to the regression equation *E*_p_ (V) = −0.058 pH + 0.692 (R^2^ = 0.999) (inset of Figure 4A). This result suggests that this biosensor obeys the Nernst equation due to the fact the slope measured is almost −59 mV/pH value, indicating that the electrochemical oxidation of UA involves the transfer of two protons (2H^+^) and two electrons (2e^−^) [39] which occur at PrGO electrode (Figure 4E). The peak current response rises sharply from pH 5.0 to 6.0 and drops when the pH increases further (Figure 4A). Thus, the chosen pH for the detection of UA is pH 6.0 due to the fact the highest peak current was obtained.

Figure 4B displays the CVs of UA at PrGO for 450 *μ*M UA for various scan rates. The results reveal that below than 100 mV/s, the anodic peak current (I_pa_) is directly proportional to the scan rate (V) (R^2^ = 0.984, Figure 4C) and the I_pa_ increases linearly with the square root of scan rate (V^1/2^) (R^2^ = 0.991, Figure 4D) above than 100 mV/s. Thus, these results indicate that the UA oxidation on PrGO electrode exhibits a process of diffusion-controlled at high scan rates and an adsorption-controlled process occurred at low scan rates [40,41,42].

### 3.4. Oxidation of UA at PrGO

**Figure 5:**
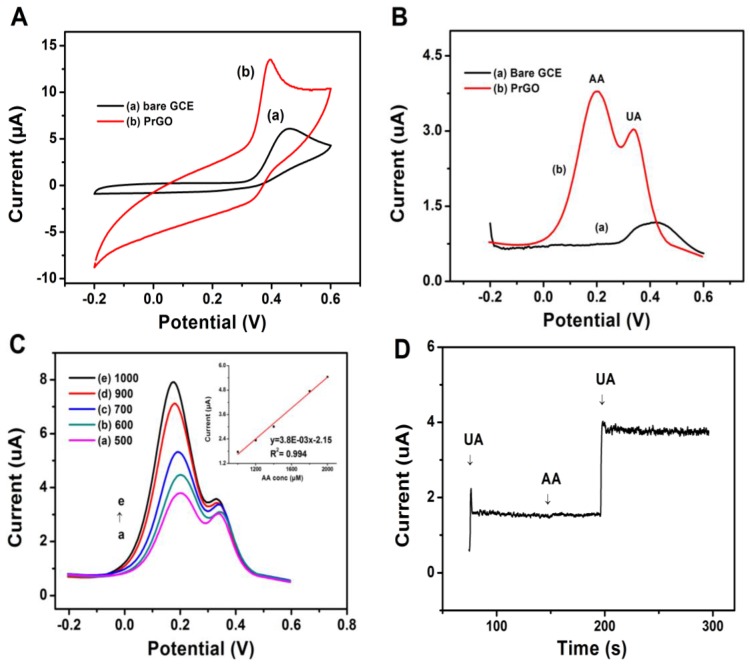
(**A**) CV of 500 μM UA at (a) bare GCE and (b) PrGO in 0.1 M phosphate buffer solution at pH 6.0 (scan rate: 50 mV/s). (**B**) DPV responses observed for (a) bare GCE and (b) PrGO electrodes in 500 μM AA and 450 μM UA in 0.1 M PBS. (**C**) DPV profiles at PrGO in PBS (pH 6.0) containing 450 μM UA and different concentrations of AA from 500–1000 μM. Inset: plots of the oxidation peak current as a function of AA concentrations. (**D**) Amperometric responses of the PrGO upon the addition of 30 μM UA, 30 μM AA, and 80 μM UA, respectively.

Figure 5A shows the CVs of 500 μM UA for a bare GCE and the PrGO. The bare GCE shows a voltammetric peak at about 0.45 V which is slightly broad implying that electron transfer kinetics occurred in slow motion, apparently due to the surface clogging of the electrode occurring as a result of the oxidation process [43]. However, an irreversible oxidation and well-defined peak at 0.36 V was observed in the PrGO which is shifted negatively by 0.09 V compared to bare GCE. This result indicates that the rate of electron transfer becomes faster which leads to the peak current and electrocatalytic active sites for the UA oxidation at PrGO composite being improved due to the synergistic effect of PEDOT and rGO.

The selectivity of PrGO in the presence of interferents was examined using DPV in a solution containing 450 μM UA and 500 μM of ascorbic acid (AA, Figure 5B). The peaks of UA and AA are apparently not distinguished very well at bare GCE due to the poor sensitivity and selectivity. Interestingly, a well separated and well-defined peak of AA and a negatively shifted UA one are obtained at the PrGO modified electrode at 0.19 and 0.38 V, respectively. As mentioned above, the electrocatalytic potential of UA obeys the Nernst equation which indicates that UA oxidation involves a two proton and two electron process (Figure 4E). Thus, this phenomenon leads to an instability of UA and the formation of UA derivatives might occur [44]. These results provide a reason why the oxidation peak of UA obtained is shifted negatively at the PrGO electrode. The selectivity of PrGO towards UA is higher compared to AA as UA exists as an anion at pH 6.0, and the solubility of UA is lower compared to AA due to the hydrophobic interaction with water [45,46]. Thus, the existence of PrGO composite could increase the number of electrocatalytic active sites between its surface and UA anion, which makes electron transfer variable and enhances the peak current of UA while the oxidation of AA is constrained [47].

In order to establish the applicability of PrGO for the selective determination of UA in the presence of AA, simultaneous changes of the AA concentrations were investigated at a fixed concentration of UA (450 μM). As shown in Figure 5C, the AA peak current is linearly proportional to its increasing concentration. The sensitivity towards the detection of AA was measured to be 3.8 × 10^−3^ μA/μM (R^2^ = 0.994). Thus, the presence of high concentrations of AA has no significant influence on the detection of UA.

Figure 5D displays an amperometric detection of the addition of 30 μM UA with subsequent addition of AA. This sensor gives a good response towards UA which indicates that no significant response change is caused by the presence of AA. Therefore, a high response towards UA is obtained without applying any enzyme or selective membrane [48]. Besides, the signal of response continues to appear after further addition of UA. This result indicates that electroactivity of PrGO does not diminish and it is highly selective for UA detection even in the presence of AA. Thus, this sensor is a good candidate for practical applications of real samples.

### 3.5. Limit of Detection

**Figure 6:**
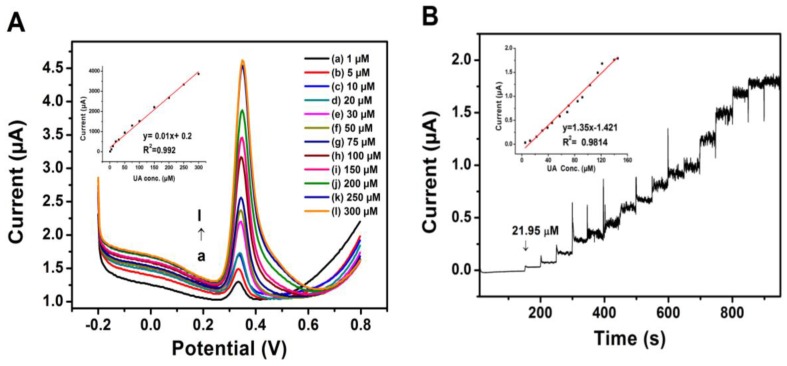
(**A**) DPV from 1 μM to 300 μM UA individually in 0.1 M PBS of pH 6.0 for PrGO. Inset: graph of the peak currents vs different concentration of UA in the linearity range of 1–300 μM. (**B**) Amperometric responses of PrGO by serial addition of 5 μM UA solutions into 0.1 M PBS (pH 6.0) solution at 0.5 V. Inset: the calibration curve for UA from 5 μM to 145 μM.

DPV and amperometry techniques were employed for the determination of the limit of detection (LOD). Based on the DPV technique, the peak current increases linearly with increasing UA concentration in the range of 1–300 μM with a linear regression equation of *I*_pa_ (μA) = 0.2 + 0.01 C_UA_(μM) (R^2^ = 0.992) and the LOD (S/N = 3) was measured to be 0.19 μM, which was calculated from typical formula of LOD = 3σ S^−1^ [42]. Figure 6B shows the response of PrGO film towards a serial addition of different concentrations of UA using the amperometry technique. The amperometry detection of UA using PrGO was operated at 0.5 V during the successive addition of 5 μM of UA in 0.1 M PBS. An increased peak current response towards the oxidation of UA across a very broad concentration range of 5–145 μM is displayed in the inset of Figure 6B. The linear response of PrGO towards UA is expressed as *I*_pa_(μA) = −1.421 + 1.35 C_UA_ (μM) (R^2^ = 0.9814) and the LOD measured is 0.32 μM (S/N = 3). This result indicates that DPV has better detection than the amperometric technique. A comparison between the proposed electrode and other modified electrodes towards detection of UA is tabulated in Table 1. The results obtained for PrGO are comparable with the reported literature results.

### 3.6. Analysis of Real Samples

The accuracy and effectiveness of the proposed sensor in a real application were tested using the standard addition method in four samples of human urine without any pretreatment. The human urine samples underwent a dilution process using 0.1 M PBS (pH 6.0). After the samples were appropriately diluted and spiked with a certain amount of UA, the analysis was performed using DPV (Table 2). The spiked sample recovery was detected within the range of 99.64% to 101.59.

In order to measure the PrGO electrode stability, repeatability and reproducibility tests were performed. The PrGO electrode offered a highly reliable current signal during 10 consecutive measurements using the same electrode in 1 mM UA with a relative standard deviation (RSD) of 3.86%. This indicates that this electrode does not experience any clogging on its surface throughout the measurements. The sensor reproducibility was measured by determining 1 mM UA with four different modified electrodes. This modified sensor depicted an excellent reproducibility with RSD value of 4.27%.

## 4. Conclusions

A PEDOT-doped reduced GO (PrGO) composite for the detection of UA was successfully prepared using the cyclic voltammetry technique. The PrGO composite provided a high peak current and low charge transfer resistance compared with bare GCE and rGO. A facile technique for the detection of UA was investigated and PrGO revealed good stability and a low detection limit with a broad linear range. PrGO composite film is not only able to serve as a sensitive and selective sensor towards UA, but it also resolved the difficulty of observing discrete UA and AA oxidation peaks, indicating that PrGO composite film is a promising candidate applicable for real applications.

## Figures and Tables

**Table 1:** Performances of uric acid from different methods and materials.

Electrode	Techniques	Detection Limit (μM)	Linear Range (μM)	Reference
GE/CFE	CV	0.13	0.194–49.68	[49]
Graphene-poly(acridine red)/GCE	DPV	0.30	0.8–150	[50]
Graphene/size-selected Pt	CV, DPV	0.05	0.05–11.9	[51]
RGO–AuNPs–CSHMs	DPV	0.70	1–300	[52]
PEDOT/Palladium	DPV	7.00	7–11	[53]
Pt/RGO	CV, DPV	0.45	10.0–130	[54]
PrGO	DPV	0.19	1–300	This work

**Table 2:** Analysis data of uric acid in real samples (*n* = 3).

Sample	Detected (μM)	Added (μM)	Found (μM)	Recovery (%)
Urine 1	242.80	100	342.44	99.64%
Urine 2	244.70	100	346.29	101.59%
Urine 3	1.36	160	161.33	99.98%
Urine 4	0.16	160	160.88	100.45%
